# A Fetal Fraction Optimized 106-Plex Digital PCR Assay for Non-Invasive Prenatal Testing of Fetal Trisomy

**DOI:** 10.3390/diagnostics16111642

**Published:** 2026-05-27

**Authors:** Songchang Chen, Xiaorui Luan, Xianling Cao, Cong Liu, Li Zhang, Wu Shang, Yiliang Zhang, Zhijie Yang, Chenming Xu

**Affiliations:** 1 Institute of Reproduction and Development, Shanghai Key Laboratory of Reproduction and Development, Obstetrics and Gynecology Hospital, Fudan University, Shanghai 200437, China; luanxiaorui7893@fckyy.org.cn (X.L.); caoxianlingling@163.com (X.C.); liucong0728@126.com (C.L.); zhangli7894@fckyy.org.cn (L.Z.); 2 Shanghai Key Laboratory of Female Reproductive Endocrine Related Diseases, Shanghai 200090, China; 3 Nanjing Pregene Biotechnology, Nanjing 210047, China; shangwu@pregene.net (W.S.);

**Keywords:** fetal fraction, super-plex, digital PCR, non-invasive prenatal testing, trisomy, high-risk singleton pregnancy

## Abstract

**Background/Objectives:** Non-invasive prenatal testing (NIPT) for fetal aneuploidy requires accurate trisomy detection together with reliable fetal fraction assessment. This study evaluated the clinical feasibility of a 106-plex digital PCR (dPCR) NIPT assay for trisomies 13, 18, and 21 with internal fetal fraction quantification. **Methods:** We consecutively recruited 470 women with high-risk singleton pregnancies. Fetal trisomies were detected using dPCR-NIPT and confirmed by invasive prenatal diagnosis. Pregnancies with negative prenatal diagnostic results were followed to birth. Analytical performance and quality control were assessed using trisomic DNA. The euploid cut-off and diagnostic performance were evaluated in two independent maternal plasma sample sets, using invasive diagnosis and clinical outcome as the reference standard. **Results:** dPCR-NIPT measured fetal fraction irrespective of fetal sex and detected trisomies at fetal fractions ≥3% using 5 ng DNA. A total of 12 of 470 plasma samples failed cell-free DNA quality control and were excluded before dPCR testing. Of the remaining 458 samples, 5 had fetal fractions below 3% and were classified as failed tests, yielding a nonreportable rate of 1.1%. Using a cut-off of 6.9 established in 103 training samples, no false-positive or false-negative trisomy calls were observed in the 350-sample testing set, corresponding to 100% sensitivity (95% confidence interval [CI], 85.18–100%) and 100% specificity (95% CI, 98.88–100%) for 23 confirmed trisomies. **Conclusions:** This proof-of-principle study supports the feasibility of fetal fraction-informed dPCR-NIPT for trisomy detection in high-risk singleton pregnancies. Larger prospective studies in average-risk and earlier-gestation populations are required.

## 1. Introduction

The most common autosomal aneuploidies are trisomy 21 (T21, Down syndrome), trisomy 18 (T18, Edwards syndrome), and trisomy 13 (T13, Patau syndrome), with estimated live-birth prevalences of 14.85, 3.24, and 1.43 per 10,000, respectively [1]. Conventional prenatal screening in the first and second trimesters combines nuchal translucency (NT) ultrasound with serum biochemical tests (SBT), including pregnancy-associated plasma protein A (PAPP-A), human chorionic gonadotropin (hCG), alpha-fetoprotein (AFP), and unconjugated estriol (uE3), used as triple or quadruple assays [2,3,4]. Because these approaches have suboptimal accuracy, positive screening results require confirmation by chorionic villus sampling (CVS) or amniocentesis (AC). These invasive procedures may cause physical discomfort and have been associated with a procedure-related pregnancy-loss risk of up to 1% [5,6,7].

NGS-based NIPT has reported pooled sensitivities of 99.3–99.5%, 93.1–97.7%, and 90.6–97.4% for T21, T18, and T13, respectively, with pooled specificity of approximately 99.9% for trisomy screening in the general population [8,9,10,11]. It is, therefore, widely used as a second-tier screen after positive conventional screening. However, first-tier NGS-NIPT can increase the number of diagnostic procedures prompted by additional findings and substantially increase costs for average-risk pregnancies [9], limiting its implementation as a universal first-tier test in many health-care settings.

dPCR is a rapid and cost-effective DNA-counting method with potential for chromosome-ratio quantification in fetal aneuploidy screening. However, the clinical performance of published dPCR-NIPT assays has generally remained below that of NGS-NIPT [12,13,14,15,16,17,18,19,20]. Key limitations include insufficient chromosomal target numbers for precise chromosome-ratio estimation and incomplete fetal fraction quality control. Low fetal fraction is a major contributor to NIPT test failure [21], and failed tests are enriched for fetal aneuploidies, particularly T18 and T13 [22]. These observations underscore the need for fetal fraction assessment within cfDNA-based screening assays.

We, therefore, conducted a prospectively registered proof-of-principle diagnostic accuracy study to evaluate a fetal fraction-optimized 106-plex dPCR-NIPT assay (FOS-dPCR-NIPT) for simultaneous fetal fraction quantification and non-invasive detection of fetal T21, T18, and T13 in high-risk singleton pregnancies.

## 2. Methods

### 2.1. Study Design and Subjects

**Figure 1:**
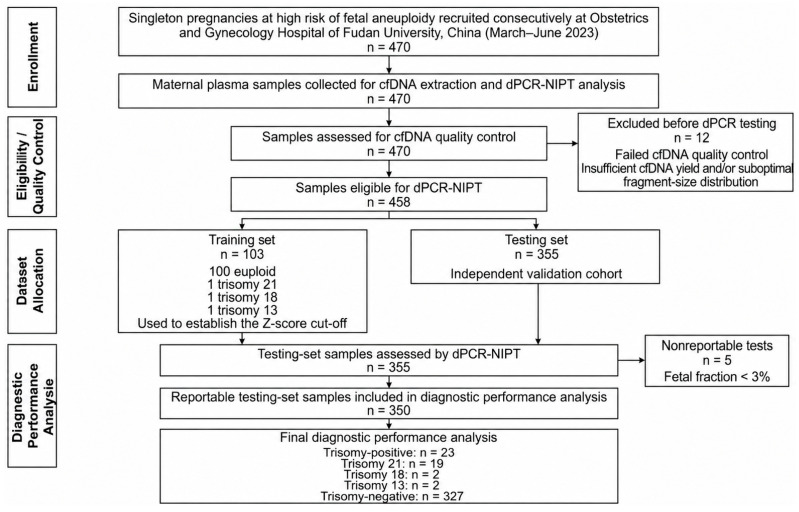
Participant flow diagram of the FOS-dPCR-NIPT study. Of 470 consecutively recruited high-risk singleton pregnancies, 12 samples failed cfDNA quality control and were excluded before dPCR testing. The remaining 458 samples were allocated to a training set (*n* = 103) or an independent testing set (*n* = 355). A total of 5 testing set samples with fetal fraction < 3% were classified as nonreportable, leaving 350 reportable testing set samples for diagnostic performance analysis. cfDNA: cell-free DNA; dPCR-NIPT: digital PCR-based non-invasive prenatal testing.

This was a prospectively registered diagnostic accuracy study of 470 consecutively recruited singleton pregnancies at high risk of fetal aneuploidy at the Obstetrics and Gynecology Hospital of Fudan University, China, from March to June 2023. Maternal plasma samples were analyzed by dPCR-NIPT, with invasive prenatal diagnosis and/or clinical outcome used as the reference standard. High-risk status was defined by advanced maternal age (≥35 years), a previous affected pregnancy, abnormal ultrasound findings, positive SBT results (T21 high risk, ≥1/270; T18 high risk, ≥1/350), and/or positive NGS-NIPT screening. Confirmatory prenatal diagnosis was performed for all pregnancies by CVS or AC with copy-number variation sequencing (CNV-seq) and karyotyping. Pregnancies with negative diagnostic results were followed to birth, and neonates were clinically assessed by their pediatricians for phenotypic features of chromosomal disease. A maternal plasma sample was collected from each participant for dPCR-NIPT. Twelve of 470 samples failed the prespecified cfDNA quality-control criteria (fragment-size distribution and/or yield) described below and were excluded before dPCR testing. These pre-dPCR QC failures reflected cfDNA yield and/or fragment-size criteria rather than fetal fraction or trisomy-calling failure. A training set of 103 patients (100 euploid, 1 T21, 1 T18, and 1 T13), confirmed by invasive diagnosis and clinical outcome, was used to establish dPCR-NIPT cut-offs. The remaining 355 patients served as an independent testing set and were assessed by dPCR-NIPT and by invasive prenatal diagnosis/clinical outcome in a double-blind manner at Pregene Biotechnology and the Obstetrics and Gynecology Hospital, respectively. Sample-size calculation was based on an estimated combined live-birth prevalence of trisomies 13, 18, and 21 of 0.2% [1] and a false-positive rate of 0.1% [8,9,10,11]. Assuming a trisomy occurrence of at least 2.5% in high-risk pregnancies, 309 subjects were required to assess diagnostic performance with 95% power and 99.9% confidence. This study was registered with the Chinese Clinical Trial Registry (ChiCTR2300069694; registration date, 23 March 2023; initial enrollment date, 25 March 2023) and approved by the Institutional Review Board of the Obstetrics and Gynecology Hospital of Fudan University in accordance with the Declaration of Helsinki (Approval No. 2021-88; Date of approval: 21 April 2021). Written informed consent was obtained from all participants and donors. The participant flow is summarized in Figure 1.

### 2.2. Sample Preparation and Assessment

After blood collection, samples were stored at 4 °C at the hospital and transferred to the laboratory for plasma preparation within 3 days according to standard hospital procedures. Plasma was separated by two-step centrifugation at 4 °C (1600 rcf for 1 min, followed by 16,000 rcf for 10 min). Genomic DNA (gDNA) was extracted from peripheral lymphocytes of adult euploid donors and from chorionic villus samples of euploid and trisomic fetuses using the DNeasy Blood and Tissue Kit (Qiagen, Valencia, CA, USA), eluted in 100 µL TE buffer, and stored at −20 °C until use. gDNA samples were sheared to approximately 160 bp using a Bioruptor Pico sonicator (Diagenode, Denville, NJ, USA) and used as reference standards for assay optimization, analytical validation, and quality control. For clinical samples, 3–10 mL peripheral blood was collected in Cell-Free DNA Storage Tubes (CWBIO, Taizhou, Jiangsu, China), and 1–4 mL plasma was obtained by sequential centrifugation and stored at −80 °C. cfDNA was extracted using the Plasma cfDNA Extraction Kit on an automated Nucleic Acid Extraction and Purification System (Pregene Biotechnology, Nanjing, Jiangsu, China), eluted in 40 µL TE buffer, and stored at −80 °C. DNA quality and quantity were evaluated using the High Sensitivity DNA Kit on a 2100 Bioanalyzer (Agilent Technologies, Santa Clara, CA, USA) and the Qubit dsDNA BR Assay Kit on a Qubit 4 Fluorometer (Thermo Fisher Scientific, Waltham, MA, USA). cfDNA samples were required to meet two criteria for dPCR testing: (1) ≥80% of fragments within the 140–180 bp range and (2) total yield ≥2 ng. To enable sex-agnostic FF estimation, cfDNA was pre-treated with methylation-sensitive restriction enzyme(s) targeting CpG sites in the RASSF1A promoter, following established RASSF1A-based protocols [23]. Digestion was performed before partitioning; each batch included a no-enzyme control and a digestion-control amplicon containing the same recognition site(s). Samples that failed digestion QC (residual maternal copies above the prespecified threshold) were re-digested or excluded. Post-digestion RASSF1A copies were used as the FO readout for FF calculation.

### 2.3. dPCR-NIPT Assay Design

FOS-dPCR-NIPT (Pregene Biotechnology, Nanjing, Jiangsu, China) was developed to target one fetal-origin (FO) marker [23], 25 regions each on chromosomes (Chr) 13, 18, and 21, and 30 regions on ChrY, yielding a 106-plex assay. Primers were designed in silico using Primer3Plus (https://www.bioinformatics.nl/cgi-bin/primer3plus/primer3plus.cgi; accessed on 10 May 2021) to target conserved regions outside common single-nucleotide polymorphisms and copy-number variants, and target specificity was verified using Primer-BLAST (https://www.ncbi.nlm.nih.gov/tools/primer-blast; 10 May 2021). All primers were inosine-intercalated, and their 5′ ends were modified with a hairpin structure to minimize interactions with probes and other primers [24]. All amplicons were shorter than 100 bp to ensure compatibility with cfDNA detection. Detection probes for Chr21, Chr18, Chr13, and ChrY/FO were labelled with HEX, FAM, CY5, and CY5.5 fluorophores, respectively, and ChrY and FO targets were distinguished by amplitude-based multiplexing [24]. All primers and probes were synthesized by Integrated DNA Technologies (IDT, San Diego, CA, USA).

### 2.4. dPCR-NIPT Workflow and Data Analysis

**Figure 2:**
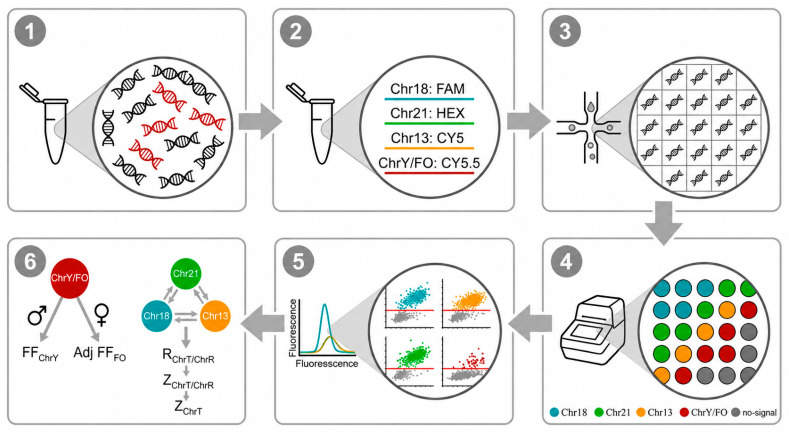
Schematic workflow of FOS-dPCR-NIPT for fetal fraction quantification and trisomy detection. The workflow includes six steps: (**1**) cfDNA extraction, 90 min; (**2**) reaction preparation, 5 min, including probes targeting Chr18/FAM, Chr21/HEX, Chr13/CY5, and ChrY/FO/CY5.5; (**3**) sample partitioning, 2 min; (**4**) PCR amplification, 150 min; (**5**) fluorescence detection and analysis, 6 min; and (**6**) fetal fraction and aneuploidy determination. Fluorescence signals are classified according to the corresponding channels, with Chr18/FAM shown in blue, Chr21/HEX in green, Chr13/CY5 in yellow/orange, ChrY/FO/CY5.5 in red, and no-signal partitions shown in grey. Fetal fraction is calculated using ChrY/FO or adjusted FO signals, and chromosome-ratio Z-scores are calculated for trisomy detection. ChrT: target chromosome; ChrR: reference chromosome; FO: fetal-origin marker; FF: fetal fraction; Adj: adjusted.

dPCR-NIPT was performed on the PRECOSMOS digital PCR system (Pregene Biotechnology, Nanjing, Jiangsu, China; Supporting Information, Figure S1a–d) according to the manufacturer’s protocol, as summarized in Figure 2. Each plate included a water control and a negative reference consisting of sheared euploid gDNA. Copy numbers (CNs) for Chr21, Chr18, and Chr13 in each PCR reaction were calculated by NeuExplorer v1.0.5.A software (Pregene Biotechnology, Nanjing, Jiangsu, China) using Poisson statistics after droplet classification. Predefined fluorescence thresholds were established from assay-development runs, water controls, and euploid negative controls. For the CY5.5 channel, two amplitude thresholds were used to distinguish the high-amplitude FO-positive droplets from the lower-amplitude ChrY/FO-positive droplet population. Thresholds were fixed before testing set analysis and were not adjusted according to clinical outcomes. FF for each sample was calculated using Equation (1) or Equation (2).

(1) $${FF}_{FO}=\frac{CN\;of\;FO}{CN\;of\;(Chr21+Chr18+Chr13)}\times 75\times 100\%$$

(2) $${FF}_{ChrY}=\frac{CN\;of\left(ChrY+FO\right)-CN\;of\;FO}{CN\;of\;(Chr21+Chr18+Chr13)}\times \frac{75}{30}\times 200\%$$

Six Z-scores (Z21/18, Z21/13, Z18/13, Z18/21, Z13/21, and Z13/18) were calculated for each sample using the euploid pregnancy population as the baseline, as previously described20. Three composite Z-scores (Z21, Z18, and Z13) were then calculated using Equation (3) or Equation (4).

If Z_ChrT/ChrR1_ > 0 and Z_ChrT/ChrR2_ > 0, then

(3) $$Z_{ChrT}=\left|Z_{ChrT/ChrR1}\times Z_{ChrT/ChrR2}\right|$$

Else

(4) $$Z_{ChrT}=-\left|Z_{ChrT/ChrR1}\times Z_{ChrT/ChrR2}\right|$$

where ChrT denotes the target chromosome being tested for fetal aneuploidy, and ChrR1 and ChrR2 denote the other two chromosomes used as references.

A combined composite Z-score (Zmax) was defined as the highest of the three composite Z-scores. The optimal cut-off was determined at the maximum Youden index in the training set, locked before testing set analysis, and then applied unchanged to the testing set to evaluate diagnostic performance. A sample with Z21, Z18, Z13, or Zmax ≥ the cut-off was classified as T21, T18, T13, or trisomy positive, respectively; samples with all composite Z-scores below the cut-off were classified as low risk for trisomy. The dPCR assays were designed, performed, and analyzed according to the dMIQE guidelines (Supplemental Material, dMIQE Checklist) [25].

### 2.5. Statistical Analysis

All statistical analyses were performed using GraphPad Prism v9.0.0 (GraphPad Software, San Diego, CA, USA). Categorical variables were compared using the χ^2^ test or Fisher’s exact test, and continuous variables were compared using the Kruskal–Wallis test or Mann–Whitney test, as appropriate. Two-sided *p* values < 0.05 were considered statistically significant. Associations between paired measurements were assessed by linear regression and Pearson correlation coefficients. Bland–Altman plots with 95% limits of agreement were used to evaluate concordance between measurements. Receiver operating characteristic (ROC) curves and areas under the ROC curve (AUCs) were used to assess diagnostic performance based on the corresponding composite Z-scores. Using invasive diagnosis and clinical outcome as the reference standard, sensitivity, specificity, positive predictive value (PPV), negative predictive value (NPV), and accuracy were calculated for dPCR-NIPT. In 106-plex runs, the FO assay shares the CY5.5 channel with the ChrY set; counts are resolved by dual-threshold gating (CN[ChrY] = CN[Y + FO]low − CN[FO]high), and the FO call and ChrY ratio thresholds were fixed in the training set to account for the modest ratio shift caused by channel sharing. Modeled PPV under lower-prevalence scenarios was calculated as PPV = sensitivity × prevalence/[(sensitivity × prevalence) + (1 − specificity) × (1 − prevalence)].

### 2.6. Role of Funders

The funders had no role in the study design; patient enrollment; data collection, analysis or interpretation; manuscript preparation; or the decision to submit the manuscript for publication.

## 3. Results

### 3.1. Analytic Validation of the FOS-dPCR-NIPT Assay

**Figure 3:**
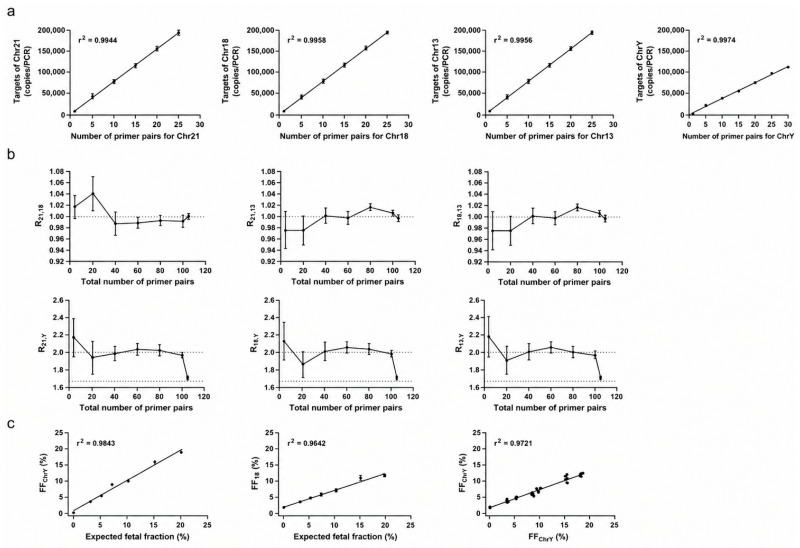
Analytical validation of multiplexity and fetal fraction quantification for FOS-dPCR-NIPT. (**a**) Linear relationship between detected target copy number and primer-pair number. (**b**) Chromosome-ratio quantification using different primer-pair numbers. Dotted lines indicate expected euploid chromosome ratios. (**c**) Linear regression of fetal fractions measured by ChrY or FO against expected fetal fractions in artificial fetal fraction mixes. Dot: mean; error bar: standard deviation; Chr: chromosome; FO: fetal-origin marker; FF_ChrY_: fetal fraction measured by ChrY; FF_FO_: fetal fraction measured by FO.

Assay multiplexity was assessed by analyzing 30 ng of sheared gDNA from adult euploid male donors with different primer-pair numbers. The detected target copy number correlated linearly with the number of primer pairs (r^2^ > 0.99; Figure 3a). As the number of primer pairs increased, the deviation of the measured chromosome ratios from the expected ratios and the corresponding standard deviations decreased (Figure 3b). These data indicate that the assay quantified multiple chromosomal targets without measurable interference and that higher multiplexity improved DNA-counting precision without increasing DNA input.

**Figure 4:**
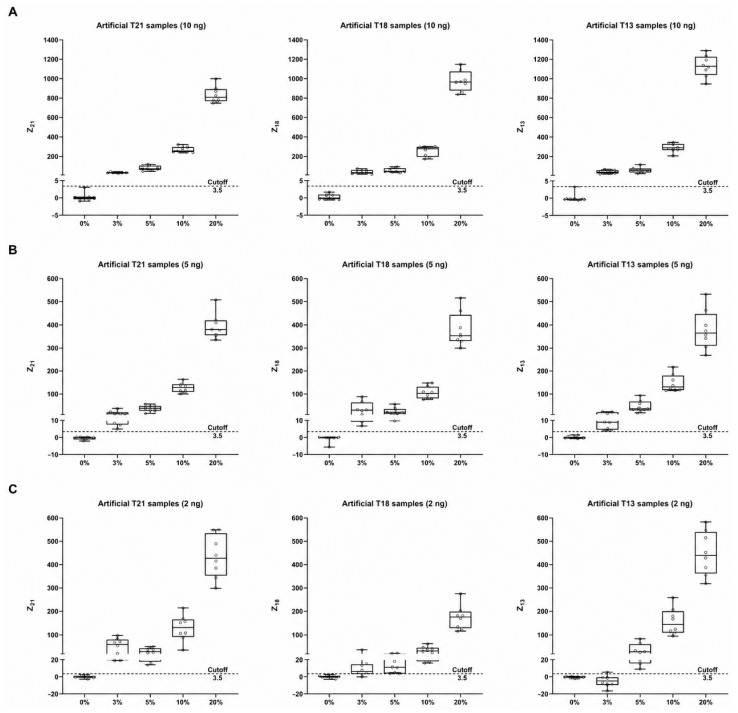
Analytical sensitivity of FOS-dPCR-NIPT using artificial trisomic DNA. Box plots show composite Z-scores (y-axis) calculated using 10 ng (**A**), 5 ng (**B**), and 2 ng (**C**) artificial trisomy DNA mixes across fetal fraction levels (x-axis). Eight replicates were tested per data point. Boxes: interquartile range; solid line: median; whiskers: range; dashed line: trisomy-detection cut-off.

Fetal-fraction quantification was evaluated using 8 ng artificial fetal fraction mixes. The expected fetal fraction correlated linearly with FFChrY (r^2^ = 0.9843) and FFFO (r^2^ = 0.9642), and the two fetal fraction estimates were also strongly correlated with each other (r^2^ = 0.9721; Figure 3c). Analytical sensitivity was then assessed using different amounts of artificial fetal fraction mixes. FFChrY remained highly correlated with the expected fetal fraction across 2–10 ng DNA (r^2^ > 0.98), whereas correlations involving FFFO decreased as DNA input declined (Supporting Information, Figure S2a–c). Thus, at least 2 ng DNA was required for reliable FFFO quantification. Analytical sensitivity for trisomy detection was evaluated using artificial trisomy samples generated by serial dilution of sheared gDNA from T21, T18, or T13 fetuses into sheared gDNA from adult euploid females. Both target copy number and positive droplet ratio (PDR; positive partitions divided by total effective partitions before Poisson correction) correlated strongly with DNA input (r^2^ > 0.97), and 2 ng or 5 ng DNA was required to achieve a PDR above 10% or 20%, respectively (Supporting Information, Figure S3a,b). At a Z-score cut-off of 3.5, the positive detection rate at 3% fetal fraction with 5–10 ng DNA was 100% for all artificial trisomy samples (Figure 4A,B), but decreased to 75% and 12.5% for T18 and T13, respectively, at 3% fetal fraction with 2 ng DNA (Figure 4C). Therefore, the lowest fetal fraction at which trisomy could be detected was 3% with 5 ng DNA or 5% with 2 ng DNA. These low-input dilution results indicate that samples with both very low fetal fraction and low cfDNA input should be interpreted conservatively as nonreportable or requiring repeat testing rather than reported unconditionally.

### 3.2. Fetal Fraction Quantification and Benchmark for Clinical Sample Testing

The number of droplets required for FOS-dPCR-NIPT to detect trisomic cell-free fetal DNA (cffDNA) with 95–99% confidence at the analytical sensitivity limit was estimated using a previously described equation [13] and ranged from 61,292 to 136,586 (Supporting Information, Table S1). Because approximately 80,000 effective droplets are routinely generated per reaction, samples with cfDNA yield > 5 ng were split into two reactions, whereas samples with 2–5 ng cfDNA were tested in one reaction to ensure sufficient droplets for trisomy detection with at least 95% confidence.

**Figure 5:**
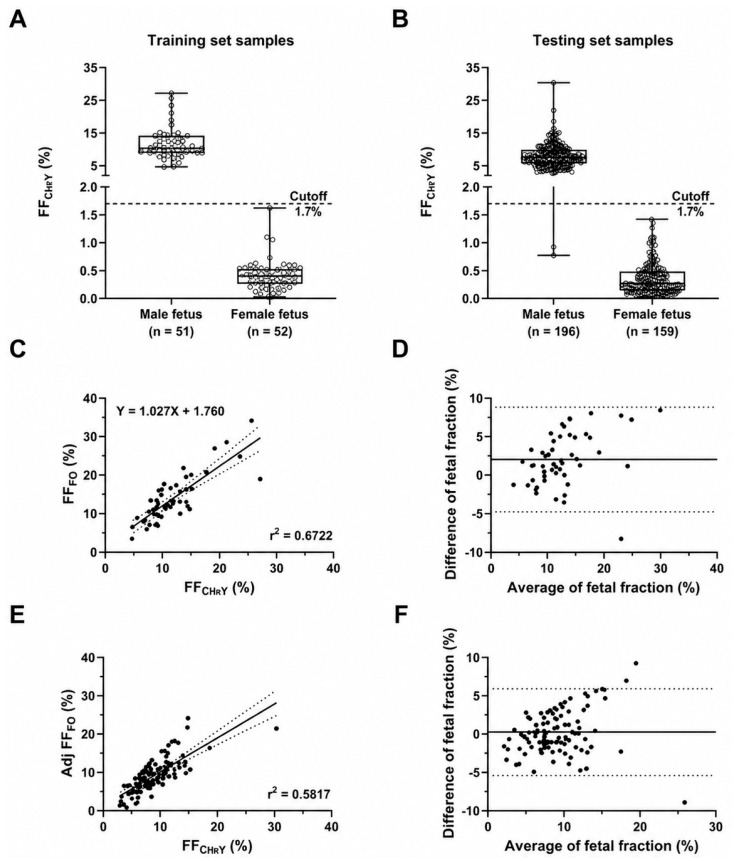
Accuracy of FOS-dPCR-NIPT for fetal-sex determination and cffDNA-fraction quantification. (**A**,**B**) Box plots of fetal fractions measured by ChrY in training (**A**) and testing (**B**) samples. Boxes: interquartile range; solid line: median; whiskers: range; dashed line: cut-off for fetal-sex determination. (**C**–**F**) Linear regression and Bland–Altman plots comparing fetal fractions measured by FO and ChrY in pregnancies with male fetuses from the training (**C**,**D**) and testing (**E**,**F**) sets. r^2^ values were derived from Pearson correlation coefficients. Adjusted FFFO values were calculated using the linear equation from (**C**). Dotted lines in linear regression plots indicate 95% confidence intervals; solid lines in Bland–Altman plots indicate mean difference for FFFO (or adjusted FFFO)—FFChrY; dotted lines in Bland–Altman plots indicate 95% limits of agreement.

The FFChrY cut-off was set at 1.7% using the 103 training samples (Figure 5A). Applying this cut-off to the 355 testing samples, the assay achieved 98.98% sensitivity (194/196; 95% CI, 96.36–99.88%) and 100% specificity (95% CI, 97.71–100%) for fetal-sex determination (Figure 5B). FFFO accuracy was evaluated in 51 training-set pregnancies with male fetuses. FFFO correlated linearly with FFChrY (r^2^ = 0.6722; Figure 5C), with a mean difference of 2.08% and 95% limits of agreement from −4.74% to 8.89% (Figure 5D). In 100 pregnancies with male fetuses randomly selected from the testing set, FFChrY and adjusted FFFO, calculated using the linear equation from Figure 5C, showed a similar linear correlation (r^2^ = 0.5817; Figure 5E), with a mean difference of 0.28% and 95% limits of agreement from −5.38% to 5.93% (Figure 5F). Therefore, fetal fraction was calculated using FFChrY for pregnancies with male fetuses and adjusted FFFO for pregnancies with female fetuses.

### 3.3. Characteristics of Study Cohort and Clinical Samples

Clinical characteristics of the study cohort and indications for invasive prenatal diagnosis are summarized in Table 1. Training and testing sets did not differ significantly, although median gestational age was marginally lower in the testing set (*p* = 0.0798). cfDNA concentration was not associated with maternal age or gestational age and did not differ significantly between the two sets, except in the second trimester, where median cfDNA concentration was lower in the testing set (*p* = 0.0361). The cffDNA fraction was positively correlated with gestational age (*p* < 0.05) and was significantly lower in the testing set (*p* < 0.05; Supporting Information, Table S2). All training-set samples and 98.59% (350/355) of testing set samples met the benchmark for trisomy detection with 95–99% confidence. Five samples with fetal fraction < 3% and insufficient cfDNA for retesting were excluded from trisomy analyses. The 12 samples excluded before dPCR testing failed cfDNA yield and/or fragment-size QC; BMI and maternal copy-number information were not systematically available for all QC-failed samples, precluding formal assessment of these clinical contributors.

### 3.4. Clinical Validation of FOS-dPCR-NIPT for Fetal Aneuploidy Detection

**Figure 6:**
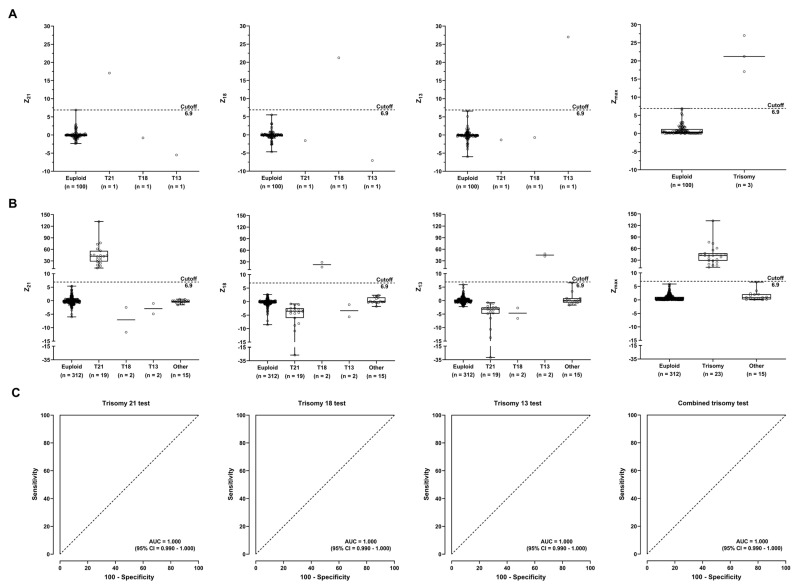
Diagnostic performance of FOS-dPCR-NIPT for trisomy detection in clinical samples. (**A**,**B**) Composite Z-score distributions in training (**A**) and testing (**B**) samples categorized by true ploidy status. Boxes: interquartile range; whiskers: range; dashed line: cut-off established from the training set. (**C**) ROC curves for trisomy-specific and combined trisomy detection in the testing set. T21: trisomy 21; T18: trisomy 18; T13: trisomy 13; ROC: receiver operating characteristic; AUC: area under the ROC curve; CI: confidence interval.

The 100 euploid samples in the training set were used to define the baseline distribution of composite Z-scores, and the cut-off was set at 6.9 (Figure 6A). Diagnostic performance was evaluated in an independent, double-blind testing set of 350 samples, with composite Z-scores calculated against the entire euploid training population. Compared with true ploidy status confirmed by invasive diagnosis and clinical outcome, FOS-dPCR-NIPT detected 19 of 19 T21, 2 of 2 T18, and 2 of 2 T13 cases, with no false-positive or false-negative trisomy calls observed (Figure 6B). The observed accuracy estimate for the three common trisomies in this high-risk testing cohort was 100% (95% CI, 98.95–100%; Table 2). AUC values were 1.000 (95% CI, 0.990–1.000) for trisomy-specific detection and for combined detection of the three trisomies (Figure 6C). Among 327 low-risk trisomy cases, invasive prenatal testing identified 15 other chromosomal abnormalities, including 10 aneuploidies, two microdeletions or microduplications, and three balanced translocations or inversions (Supporting Information, Table S3), which were outside the intended scope of FOS-dPCR-NIPT.

Stratified analyses by clinical characteristics showed that the three common trisomies were significantly associated with advanced maternal age (*p* < 0.01) and lower gestational age (*p* = 0.0170). These factors did not differ significantly between aneuploidy-positive and aneuploidy-negative cases (Supporting Information, Table S4).

### 3.5. Comparison of FOS-dPCR-NIPT and NGS-NIPT for Fetal Aneuploidy Screening in Clinical Settings

Among the 66 testing set samples with both FOS-dPCR-NIPT and NGS-NIPT results, diagnostic performance was evaluated using the same reference standard (Supporting Information, Table S5). Both methods detected all 12 trisomy cases, yielding 100% sensitivity (95% CI, 73.54–100%), whereas NGS-NIPT reported one false-positive T21 result and two false-positive T13 results. For broader fetal aneuploidy screening, NGS-NIPT reported 32 aneuploidy cases, including two false negatives and 14 false positives, whereas FOS-dPCR-NIPT detected 12 of 20 aneuploidy cases with no false positives (Supporting Information, Table S6). Sensitivity and specificity for detecting the three common trisomies did not differ significantly between the two methods. For all aneuploidies, NGS-NIPT showed 30.00% higher sensitivity (*p* = 0.0313), reflecting its broader chromosomal coverage, but 30.43% lower specificity (*p* = 0.0001) than FOS-dPCR-NIPT. Overall, AUC-based diagnostic performance did not differ significantly between the two methods for trisomy or aneuploidy detection (Table 3).

Because T21 is the most commonly screened autosomal trisomy and T13 and T18 have low fetal survival rates, the modeled clinical benefit of common-trisomy NIPT is driven largely by T21 detection. We, therefore, assessed the costs and health outcomes of seven prenatal screening strategies for T21 (Supporting Information, Figure S4), using variables from the present and previous studies and data from local official statistics (Supporting Information, Table S7). Amplicon context information for all 106 assays, including the reference genome build, chromosomal coordinates, and approximately 30 bp flanking sequences, is provided in Supporting Information, Table S8. Cost-effectiveness simulation results for the seven T21 screening strategies in a cohort of 10,000 pregnant women are shown in Table 4. FOS-dPCR-NIPT was more cost-effective when modeled at maximum sensitivity, whereas NGS-NIPT was more cost-effective when FOS-dPCR-NIPT was modeled at maximum specificity.

## 4. Discussion

In this study, FOS-dPCR-NIPT measured fetal fraction irrespective of fetal sex and detected the three common trisomies at fetal fractions ≥ 3%, with no false-positive or false-negative trisomy calls in an independent high-risk testing cohort. Preliminary comparison with NGS-NIPT suggested complementary performance profiles: FOS-dPCR-NIPT was highly specific for targeted common trisomies, whereas NGS-NIPT provided broader chromosomal coverage and higher sensitivity for all aneuploidies.

Previous studies have shown that dPCR-NIPT can detect T21, T18, or T13 at fetal fractions ≥ 5% with >90% accuracy by using cffDNA enrichment, multiplexed assay design, and/or larger partition numbers 16–18,20. A recent proof-of-concept study reported a 222-plex dPCR-NIPT assay that detected 4% T21 in 11 ng artificial DNA mixes, but clinical diagnostic performance was not evaluated19. To our knowledge, the 106-plex FOS-dPCR-NIPT described here is among the highest-plex dPCR-NIPT assays evaluated in clinical samples. It measured fetal fraction in both fetal sexes, and fetal fraction increased with gestational age, consistent with previous reports [26,27,28]. In the high-risk testing cohort, no false-positive or false-negative trisomy call was observed for T21, T18, or T13, and the nonreportable rate due to low fetal fraction was 1.1% (5/458), comparable with reported NGS-NIPT failure rates [21,22]. The association between advanced maternal age and the three common trisomies was also consistent with previous studies [29,30,31,32,33].

Compared with NGS-NIPT, FOS-dPCR-NIPT has a simpler workflow that does not require library preparation or intensive computation and can generate results within one day for up to 96 samples per plate run. The assay also does not generate unsolicited sequence information. These features may make FOS-dPCR-NIPT attractive for common-trisomy-focused workflows in routine clinical laboratories after larger prospective validation. This study also reinforces the need to measure fetal fraction as part of NIPT quality control, because samples below the fetal fraction threshold should be retested rather than reported as negative. The assay combines MIQE-compliant primer/probe design, a 5′ hairpin tail to support multiplexing, and dual fetal fraction estimation using ChrY and FO markers. Requiring concordant evidence from two independent chromosome-ratio Z-scores and using their product as the decision variable may reduce variability from assay CV and locus-level copy-number differences, thereby improving specificity without compromising sensitivity. These design features warrant prospective validation across gestational ages and clinical risk strata.

Future work should evaluate assay expansion to sex chromosome aneuploidies and selected microdeletion syndromes, such as 22q11.2, 5p, 15q11-q13, and 1p36 deletions. Performance of an expanded assay may be further improved by higher-plex design on a high-partition-capacity dPCR system.

The main strength of this study is the integration of universal fetal fraction quantification with high-plex dPCR-based trisomy detection. The assay detected trisomy using 5 ng DNA at cffDNA fractions as low as 3%, an input obtainable from a typical 5–10 mL blood draw, thereby avoiding potential chromosome-ratio skewing introduced by cffDNA enrichment. The two male fetuses incorrectly categorized by our assay as female were diagnosed by invasive testing as monosomy X mosaicism with partial deletion of chromosome Y, indicating the limitation of the current assay for such cases because of limited Y-chromosome coverage. The assay uses absolute molecular counting with short amplicons in a single reaction, which is compatible with low-input, fragmented cfDNA and minimizes preparative bias. Concordant chromosome-ratio statistics reduce locus-level variability and general assay CV. Orthogonal fetal fraction estimates (ChrY and sex-agnostic FO markers) support interpretation across fetal sex and sample heterogeneity. The cut-offs were fixed in the training set by maximizing the Youden index for the product of two chromosome-ratio Z-scores, and this single operating point was applied unchanged to the validation cohort. The minimum input (≥5 ng) and fetal fraction (≥3%) reflect analytical constraints: at approximately 5 ng (~1.5 × 10^3^ haploid genome equivalents), Poisson counting provides stable chromosome-ratio variance, whereas FF < 3% reduces the expected fetal signal below this precision and increases the risk of discordance, supporting retesting.

Although this study was powered for diagnostic assessment in a high-risk cohort, the number of trisomy-positive cases was limited, particularly for T18 and T13, with two cases each. The findings should, therefore, be interpreted as proof-of-principle evidence rather than definitive population-level clinical validation. PPV is prevalence-dependent; at an assumed average-risk trisomy prevalence of 0.2%, modeled PPV would be approximately 15.2% using the lower bound of the observed specificity confidence interval and approximately 66.7% at 99.9% specificity. Larger prospective average-risk studies are needed to define real-world PPV, specificity, and no-call rates.

Additional limitations include the predominance of second-trimester samples (94%), the need to validate performance in earlier gestation when fetal fractions may be lower, and the preliminary nature of the comparison with NGS-NIPT. The Zmax cut-off of 6.9 was derived from the training set and applied unchanged to the testing set; it should, therefore, be considered a provisional operating point until externally validated in larger cohorts. As with all cfDNA-based screening tests, both dPCR- and NGS-based NIPT may be affected by rare biological factors, such as confined placental mosaicism or maternal chromosomal abnormalities, that are not fully resolved by fetal fraction QC alone.

In conclusion, this fetal fraction-informed 106-plex dPCR assay showed promising proof-of-principle performance for targeted non-invasive detection of common fetal trisomies in high-risk singleton pregnancies. Its short turnaround time, simple workflow, and lower cost support further clinical evaluation. Larger prospective studies with external validation, including first-trimester and average-risk cohorts and head-to-head comparison with NGS-NIPT, are required before conclusions can be drawn about population-wide implementation.

## Figures and Tables

**Table 1:** Clinical characteristics of the study cohort.

Characteristics	All (*n* = 458)	Training Set (*n* = 103)	Testing Set (*n* = 355)	*p* Value
Maternal age, years				
Median (IQR)	33 (30–36)	33 (31–37)	33 (30–36)	0.8664 ^a^
<35	266 (58.08%)	64 (62.14%)	202 (56.90%)	0.3658 ^b^
≥35	192 (41.92%)	39 (37.86%)	153 (43.10%)	
Gestational age, weeks				
Median (IQR)	18.29 (17.29–22.57)	18.43 (17.43–23.00)	18.29 (17.14–22.43)	0.0798 ^a^
First trimester (11–13)	11 (2.40%)	3 (2.91%)	8 (2.25%)	0.3124 ^c^
Second trimester (14–27)	431 (94.11%)	94 (91.26%)	337 (94.93%)	
Third trimester (28–40)	16 (3.49%)	6 (5.83%)	10 (2.82%)	
Clinical indications				
Positive SBT ^d^, (*n* = 38)	36 (94.74%)	8 (88.89%)	28 (96.55%)	0.8628 ^c^
Abnormal ultrasound	188 (41.05%)	44 (42.72%)	144 (40.56%)	
Previous affected pregnancy	92 (20.09%)	18 (17.48%)	74 (20.85%)	
Positive NGS-NIPT ^d^, (*n* = 82)	42 (51.22%)	8 (50.00%)	34 (51.52%)	
Invasive diagnostic tests				
CVS	11 (2.40%)	3 (2.91%)	8 (2.25%)	0.7165 ^b^
AC	447 (97.60%)	100 (97.09%)	347 (97.75%)	

IQR, interquartile range; SBT, serum biochemical test; NGS-NIPT, next-generation sequencing-based non-invasive prenatal test; CVS, chorionic villus sampling; AC, amniocentesis. ^a^ Mann–Whitney test. ^b^ Fisher’s exact test. ^c^ χ^2^ test. ^d^ SBT and/or NGS-NIPT were not performed for every pregnancy; only pregnancies with reportable results were included.

**Table 2:** Diagnostic performance of FOS-dPCR-NIPT for the indicated aneuploidy in 350 testing set plasma samples.

Aneuploidy Identified by FOS-dPCR-NIPT	True Ploidy Status Confirmed by Invasive Diagnosis and Clinical Outcome							
	Trisomy 21		Trisomy 18		Trisomy 13		All Three Trisomies	
	Positive	Negative	Positive	Negative	Positive	Negative	Positive	Negative
Positive	19	0	2	0	2	0	23	0
Negative	0	331	0	348	0	348	0	327
**Diagnostic Performance**	**Trisomy 21 Detection**		**Trisomy 18 Detection**		**Trisomy 13 Detection**		**Combined Trisomy Detection**	
AUC (95% CI)	1.000 (0.990–1.000)		1.000 (0.990–1.000)		1.000 (0.990–1.000)		1.000 (0.990–1.000)	
Sensitivity, % (95% CI)	100.00 (82.35–100.00)		100.00 (15.81–100.00)		100.00 (15.81–100.00)		100.00 (85.18–100.00)	
Specificity, % (95% CI)	100.00 (98.89–100.00)		100.00 (98.95–100.00)		100.00 (98.95–100.00)		100.00 (98.88–100.00)	
PPV, % (95% CI)	100.00		100.00		100.00		100.00	
NPV, % (95% CI)	100.00		100.00		100.00		100.00	
Accuracy, % (95% CI)	100.00 (98.95–100.00)		100.00 (98.95–100.00)		100.00 (98.95–100.00)		100.00 (98.95–100.00)	

AUC, area under the receiver operator characteristic curve; PPV, positive predicative value; NPV, negative predicative value; CI, confidence interval.

**Table 3:** Diagnostic performance comparison for trisomy and aneuploidy detection in 66 testing set plasma samples.

Diagnostic Performance ^a^	Combined Trisomy Detection			Combined Aneuploidy Detection		
	FOS-dPCR-NIPT	NGS-NIPT	*p* Value	FOS-dPCR-NIPT	NGS-NIPT	*p* Value
AUC (95% CI)	1.000 (0.946–1.000)	0.972 (0.899–0.997)	0.0774 ^b^	0.800 (0.683–0.888)	0.798 (0.681–0.887)	0.9724 ^b^
Sensitivity, % (95% CI)	100.00 (73.54–100.00)	100.00 (73.54–100.00)	NA	60.00 (36.05–80.88)	90.00 (68.30–98.77)	0.0313 ^c^
Specificity, % (95% CI)	100.00 (93.40–100.00)	94.44 (84.61–98.84)	0.2500 ^c^	100.00 (92.29–100.00)	69.57 (54.25–82.26)	0.0001 ^c^

NGS-NIPT, next-generation sequencing-based non-invasive prenatal test; AUC, area under the receiver operator characteristic curve; CI, confidence interval; NA, not applicable. ^a^ Diagnostic performance of FOS-dPCR-NIPT and NGS-NIPT was compared to invasive diagnosis and clinical outcome as the gold standard. ^b^ Nonparametric pairwise comparison of AUC for all variables. ^c^ McNemar test was performed with two-sided *p* values.

**Table 4:** Cost-effectiveness analysis results of seven prenatal screening strategies for trisomy 21.

	Current Strategy in China	Hybrid Strategy		Primary cffDNA Screening		Contingent Strategy	
	Age + SBT + NT + NGS-NIPT	Age + NGS-NIPT	Age + dPCR-NIPT	Universal NGS-NIPT	Universal dPCR-NIPT	SBT + NT + NGS-NIPT	dPCR-NIPT + NGS-NIPT
dPCR-NIPT performance: sen (100%)/spe (98.89%)							
Health outcomes, *n*							
T21 cases terminated (effectiveness)	8.85	8.84	8.88	11.2	11.25	7.88	10.64
Live-birth T21 cases	4.48	4.49	4.46	2.71	2.67	5.21	3.14
T21 miscarriages	1.49	1.5	1.49	0.9	0.89	1.74	1.05
Non-T21 miscarriages	1527.18	1527.73	1527.73	1527.73	1527.73	1527.73	1527.73
PRLs	3.62	0.03	0.08	0.04	0.3	0.02	0.03
Live-birth healthy neonates	8454.38	8457.42	8457.37	8457.42	8457.16	8457.42	8457.42
Total	10,000	10,000	10,000	10,000	10,000	10,000	10,000
Cost, $	11,639,616.83	10,924,392.33	10,792,998.69	12,019,623.09	11,293,942.39	10,731,231.56	11,242,346.31
CER ^a^	1,314,943.61	1,236,411.55	1,215,432.85	1,073,575.43	1,003,714.88	1,362,289.18	1,057,000.20
Incremental cost, $	908,385.27	193,160.77	61,767.13	1,288,391.53	562,710.83	-	511,114.75
Incremental effectiveness, *n*	0.97	0.96	1	3.32	3.37	-	2.76
ICER ^b^	932,205.83	201,584.78	61,606.28	388,241.74	166,739.51	-	185,271.41
dPCR-NIPT performance: sen (82.35%)/spe (100%)							
Health outcomes, *n*							
T21 cases terminated (effectiveness)	8.85	8.84	7.31	11.2	9.27	7.88	8.76
Live-birth T21 cases	4.48	4.49	5.64	2.71	4.17	5.21	4.55
T21 miscarriages	1.49	1.5	1.88	0.9	1.39	1.74	1.52
Non-T21 miscarriages	1527.18	1527.73	1527.73	1527.73	1527.73	1527.73	1527.73
PRLs	3.62	0.03	0.02	0.04	0.03	0.02	0.03
Live-birth healthy neonates	8454.38	8457.42	8457.42	8457.42	8457.42	8457.42	8457.42
Total	10,000	10,000	10,000	10,000	10,000	10,000	10,000
Cost, $	11,639,616.83	10,924,392.33	10,779,053.86	12,019,623.09	11,218,927.59	10,731,231.56	11,220,975.31
CER ^a^	1,314,943.61	1,236,411.55	1,474,028.51	1,073,575.43	1,210,744.59	1,362,289.18	1,281,106.14
Incremental cost, $	908,385.27	193,160.77	47,822.29	1,288,391.53	487,696.03	-	489,743.74
Incremental effectiveness, *n*	0.97	0.96	−0.56	3.32	1.39	-	0.88
ICER ^b^	932,205.83	201,584.78	−84,685.81	388,241.74	351,166.94	-	555,601.57

SBT, serum biochemical test; NT, nuchal translucency; NGS-NIPT, next-generation sequencing-based non-invasive prenatal test; dPCR-NIPT, digital PCR-based non-invasive prenatal test; sen, sensitivity; spe, specificity; T21, trisomy 21; PRL, procedure-related fetus loss caused by invasive diagnosis; CER, cost-effectiveness ratio; ICER, incremental cost-effectiveness ratio. ^a^ CER is defined as the cost per case of T21 that is averted. ^b^ ICER is calculated between each strategy and the cheapest strategy as ICER = Δcost/Δeffectiveness.

## Data Availability

The original contributions presented in this study are included in the Supplementary Materials. Further inquiries can be directed to the corresponding authors.

## Supplementary Materials

The following supporting information can be downloaded at: https://www.mdpi.com/article/10.3390/diagnostics16111642/s1. dMIQE Checklist. Figure S1: Structure of the microfluidic chip and the generated droplets. (a) The sandwich structure of the chip (93 mm × 23 mm × 17.5 mm): the top layer has 8 separate inlets for adding oil (4 larger inlets) and PCR reaction mix (4 smaller inlets), the middle layer contains microfluidic channels for generating around 100,000 water-in-oil emulsion droplets per sample with an effective droplet ratio of above 80%, and the bottom layer contains outlets for automatic dispense of droplets into the 96-well PCR plate. (b) The schematic of the middle layer containing separate inlets/outlets (white dots) and microfluidic channels (white lines). (c) The magnified view of the intersection of the water and oil channels where emulsion droplets are generated. (d) Generated emulsion droplets with an average volume of 0.6 nl form a single layer under the brightfield microscope; Figure S2: Analyses of fetal fraction quantification sensitivity with different amount of input DNA. 10 ng (a), 5 ng (b) and 2 ng (c) of artificial trisomy samples were tested, in 24 replicates for each of 3%, 5%, 10% and 20% fetal fraction of 2–10 ng DNA, and in 8 replicates for 0% fetal fraction of 10 ng DNA. r^2^ values were derived from the Pearson correlation coefficient. Dot: mean; error bar: standard deviation; FF_ChrY_: fetal fraction measured by ChrY; FFFO: fetal fraction measured by FO; Figure S3: Linearity of DNA with target copy number (a) and positive droplet ratio (b). A total of 2 ng, 5 ng, and 10 ng of sheared gDNA from adult euploid females were tested in 8 replicates per data point. r^2^ values were derived from the Pearson correlation coefficient. Dot: mean; error bar: standard deviation; PDR: positive droplet ratio; Figure S4: Seven prenatal screening strategies for trisomy 21. The type of testing is indicated in the boxes, and results are listed next to the connecting lines. SBT includes ꞵ-human chorionic gonadotropin (ꞵ-hCG), alpha-fetoprotein (AFP), and unconjugated estriol (uE3) as a triple assay, with a cut-off of ≥1/270 as T21 high-risk or a cut-off between 1/1000-1/270 as T21 intermediate-risk. NIPT includes either NGS-NIPT or dPCR-NIPT. Invasive diagnosis includes either chorionic villus sampling or amniocentesis. SBT, serum biochemical test; NT, nuchal translucency; NGS-NIPT, next-generation sequencing-based non-invasive prenatal testing; dPCR-NIPT, digital PCR-based non-invasive prenatal testing; cffDNA, cell-free fetal DNA; Table S1: Estimation of droplet number required for FOS-dPCR-NIPT; Table S2: Characteristics of 458 cfDNAs in association with maternal age and gestational age; Table S3: Characteristics and test results of 38 testing set subjects with confirmed chromosomal anomalies; Table S4: Association of fetal ploidy status with clinical characteristics for 350 testing set subjects; Table S5: Results of NGS-NIPT and FOS-dPCR-NIPT compared with invasive diagnosis and clinical outcome for 66 testing set plasma samples; Table S6: Diagnostic performance of FOS-dPCR-NIPT and NGS-NIPT for the indicated aneuploidy in 66 testing set plasma samples; Table S7: Variables in the cost-effectiveness model; Table S8: Locations and sequence. References [34,35,36,37] are cited in the supplementary materials.

## Abbreviations

NIPT	non-invasive prenatal testing
dPCR	digital PCR
CI	confidence interval
NGS	next-generation sequencing
NT	nuchal translucency
SBT	serum biochemical tests
PAPP-A	pregnancy associated plasma protein A
hCG	human chorionic gonadotropin
AFP	alpha-fetoprotein
uE3	unconjugated estriol
CVS	chorionic villus sampling
AC	amniocentesis
FOS-dPCR-NIPT	dPCR-NIPT with fetal fraction optimization
cfDNA	cell-free DNA
ChiCTR	Chinese Clinical Trial Registry
gDNA	Genomic DNA
CN	copy number
ROC	receiver operating characteristic
AUC	area under the ROC curve
PPV	positive predictive value
NPV	negative predictive value
