# Supplementary material for: A Fetal Fraction Optimized 106-Plex Digital PCR Assay for Non-Invasive Prenatal Testing of Fetal Trisomy
Source: Diagnostics (Basel). 2026 May 27;16(11):1642. doi: 10.3390/diagnostics16111642 (PMC13256678; doi:10.3390/diagnostics16111642)
Supplement: Supplementary file 1 [file diagnostics-16-01642-s001.zip › Supporting Information.pdf]

Supporting Information

This supporting information file includes:

Figures S1-S4

Tables S1-S7

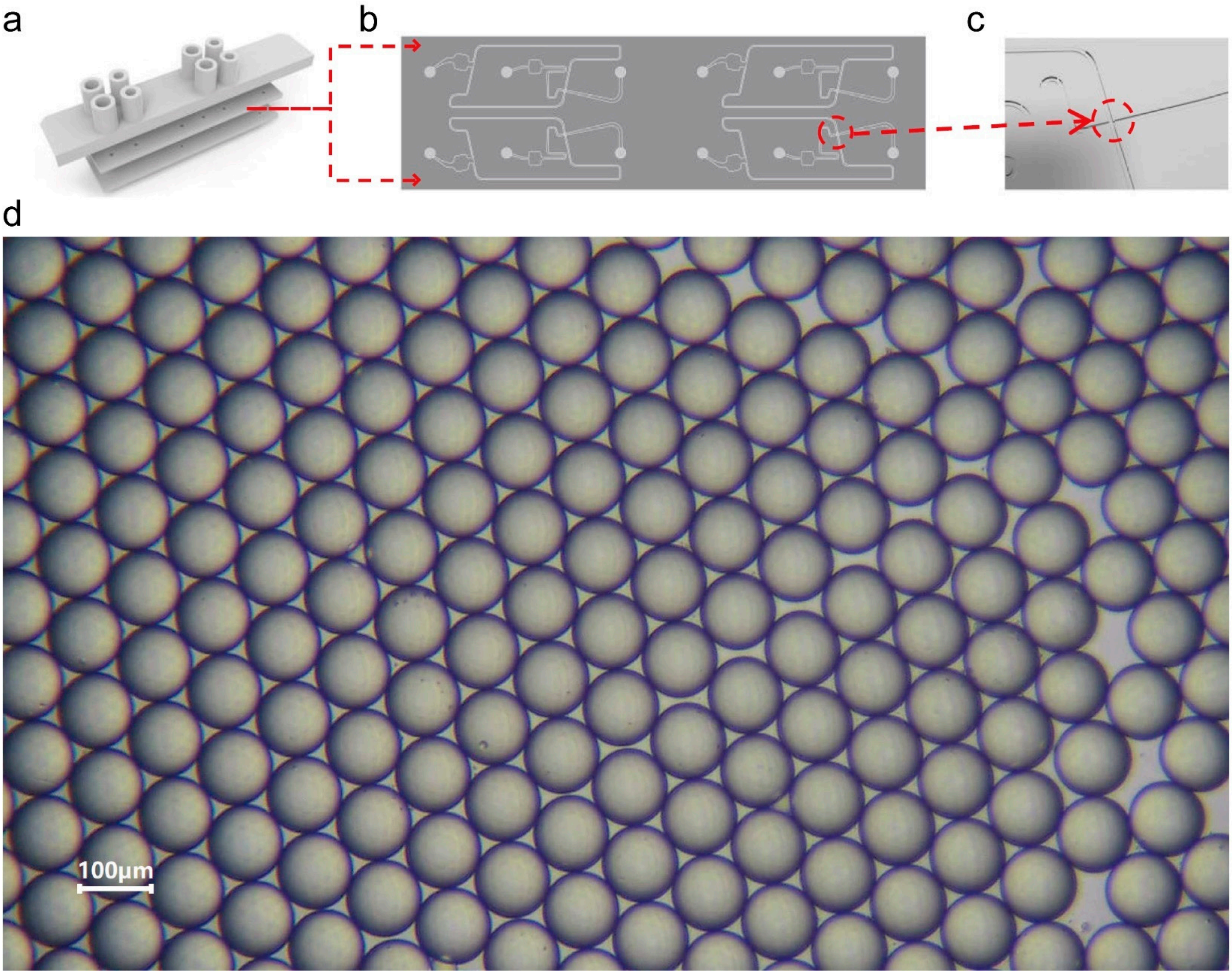

Figure S1 Structure of the microfluidic chip and the generated droplets. (a) The sandwich structure of the chip (93 mm x 23 mm x 17.5 mm): the top layer has 8 separate inlets for adding oil (4 larger inlets) and PCR reaction mix (4 smaller inlets), the middle layer contains microfluidic channels for generating around 100,000 water-in-oil emulsion droplets per sample with an effective droplet ratio of above 80%, and the bottom layer contains outlets for automatic dispense of droplets into the 96-well PCR plate. (b) The schematic of the middle layer containing separate inlets/outlets (white dots) and microfluidic channels (white lines). (c) The magnified view for the intersection of the water and oil channels where emulsion droplets are generated. (d) Generated emulsion droplets with an average volume of 0.6 nl form a single layer under the brightfield microscope.

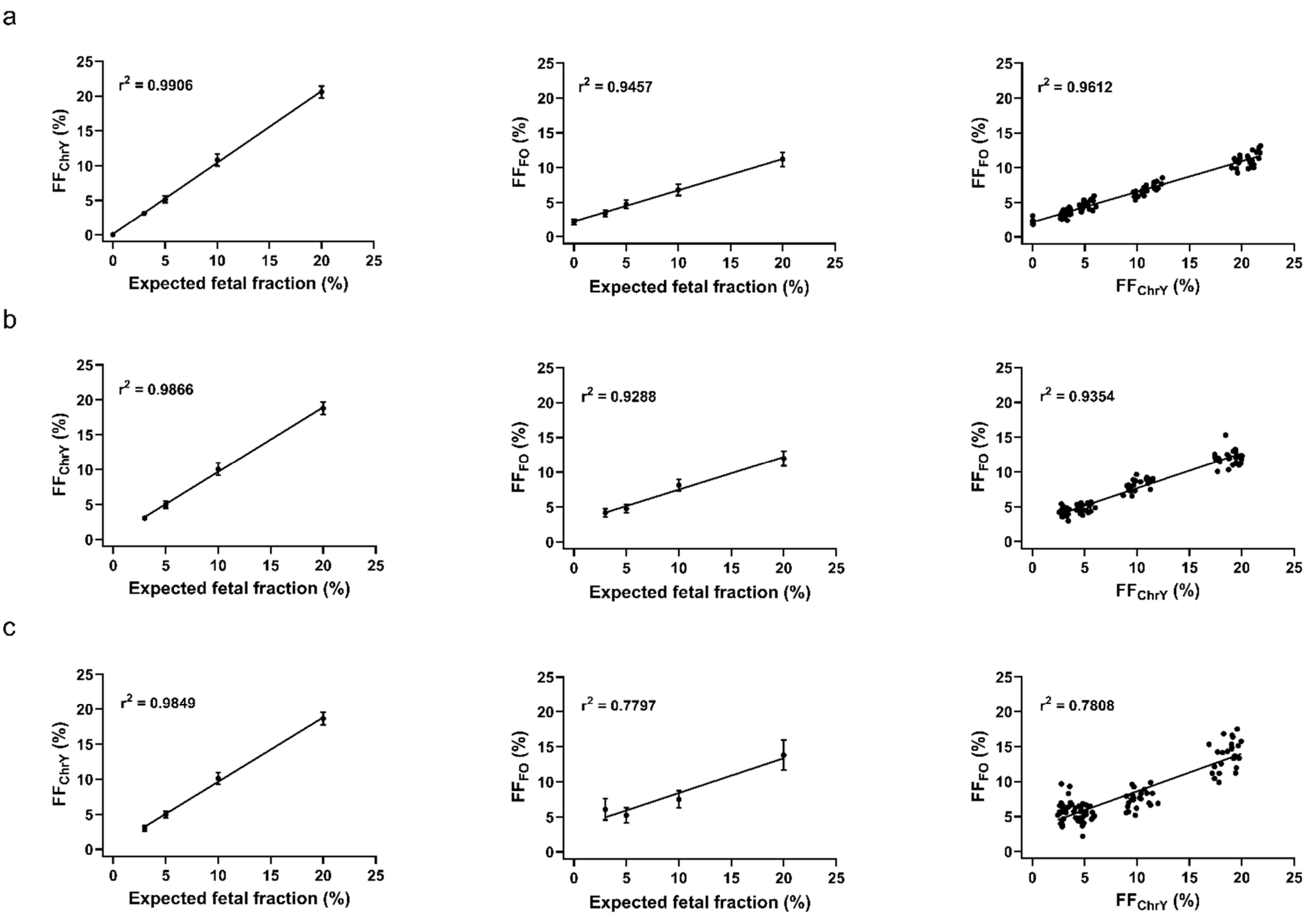

Figure S2 Analyses of fetal fraction quantification sensitivity with different amount of input DNA. 10 ng (a), 5 ng (b) and 2 ng (c) of artificial trisomy samples were tested, in 24 replicates for each of 3%, 5%, 10% and 20% fetal fraction of 2-10 ng DNA, and in 8 replicates for 0% fetal fraction of 10 ng DNA. r^2 values were derived from Pearson correlation coefficient. Dot: mean; error bar: standard deviation; FF_{ChrY}: fetal faction measured by ChrY; FF_{FO}: fetal faction measured by FO.

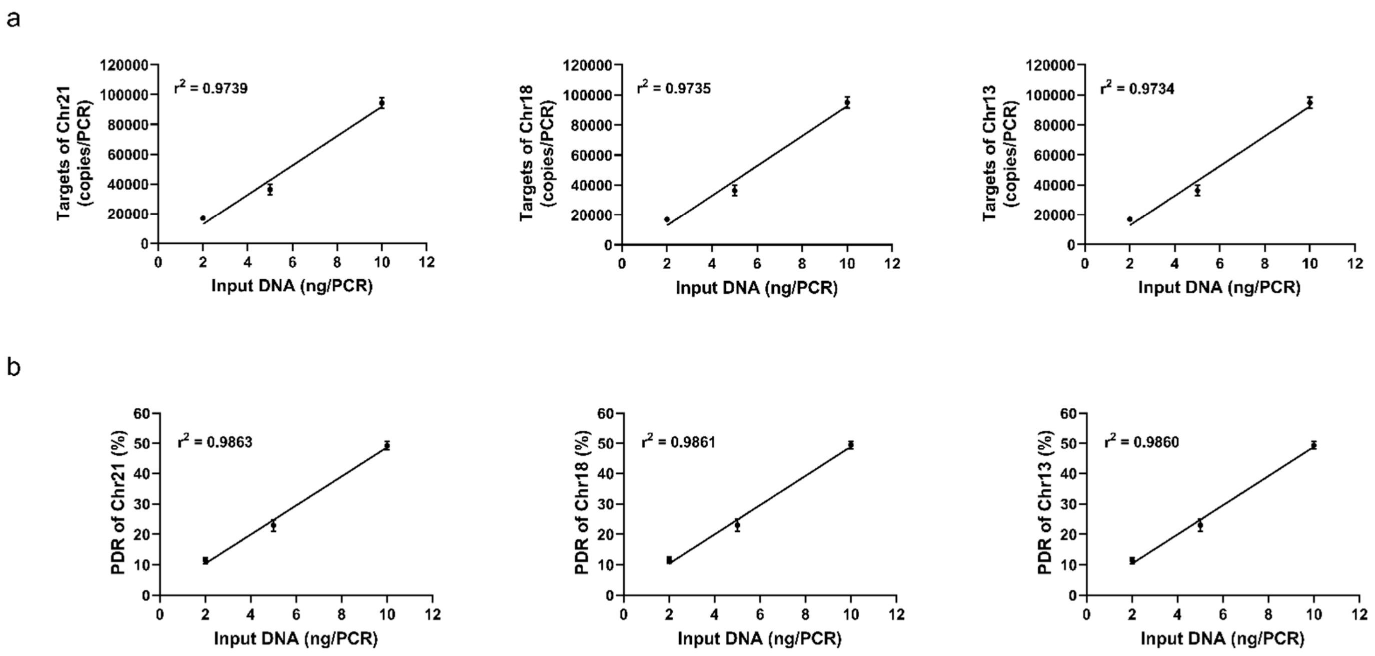

Figure S3 Linearity of DNA with target copy number (a) and positive droplet ratio (b). 2 ng, 5 ng and 10 ng of sheared gDNA from adult euploid females were tested in 8 replicates per data point. r^2 values were derived from Pearson correlation coefficient. Dot: mean; error bar: standard deviation; PDR: positive droplet ratio.

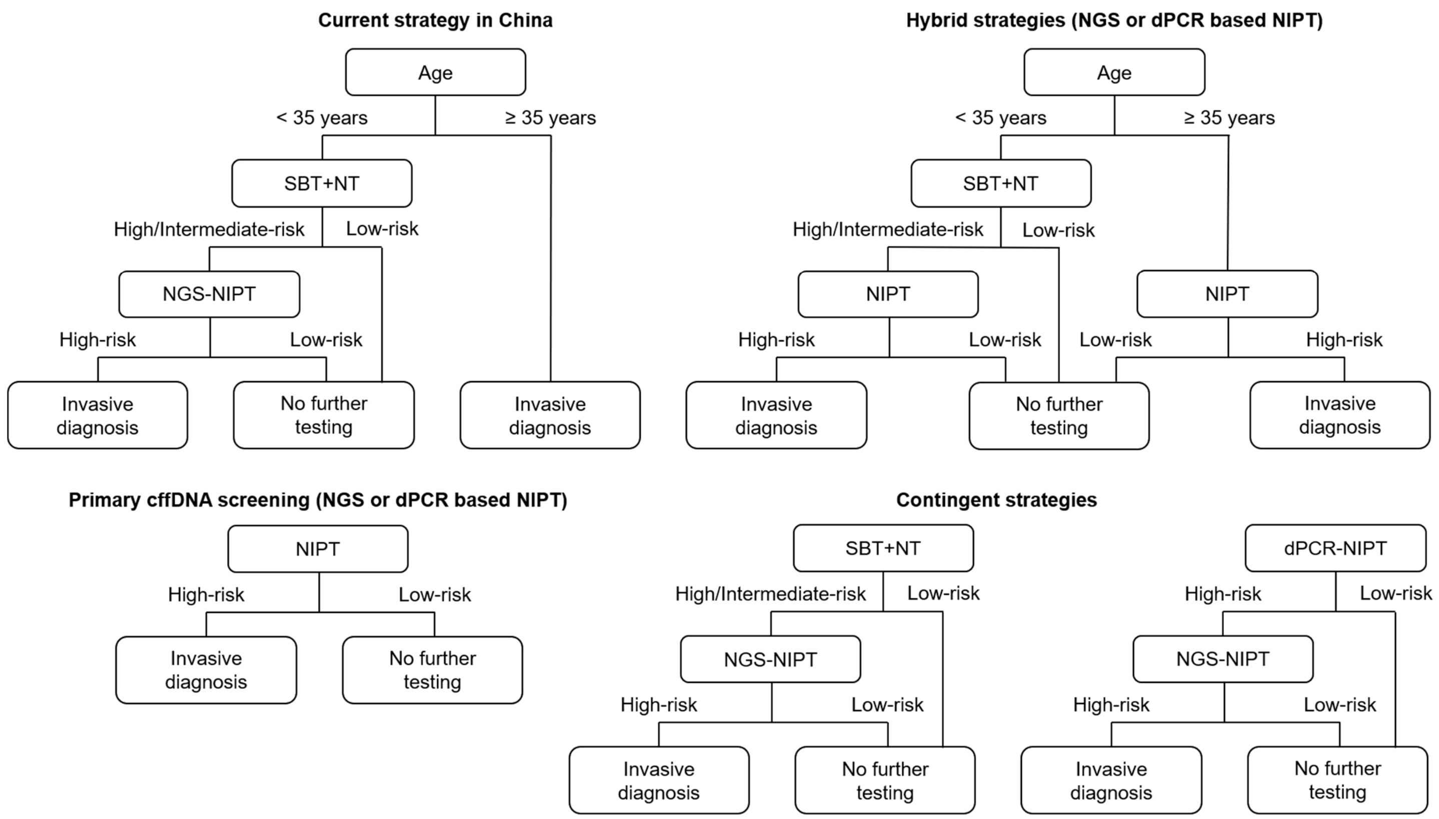

Figure S4: Seven prenatal screening strategies for trisomy 21. Type of testing is indicated in the boxes and results are listed next to the connecting lines. SBT includes β-human chorionic gonadotropin (β-hCG), alpha-fetoprotein (AFP), and unconjugated estriol (uE3) as triple assay, with a cut-off of ≥ 1/270 as T21 high-risk or a cut-off between 1/1000-1/270 as T21 intermediate-risk. NIPT includes either NGS-NIPT or dPCR-NIPT. Invasive diagnosis includes either chorionic villus sampling or amniocentesis. SBT, serum biochemical test; NT, nuchal translucency; NGS-NIPT, next generation sequencing based non-invasive prenatal testing; dPCR-NIPT, digital PCR based non-invasive prenatal testing; cffDNA, cell-free fetal DNA.

Table S1: Estimation of droplet number required for FOS-dPCR-NIPT.

Positive droplet ratio	cffDNA fraction			
	3%	5%	10%	20%
Trisomy detection with 95% confidence				
10%	166,949	61,292	16,080	4,412
20%	78,828	28,940	7,593	2,084
Trisomy detection with 99% confidence				
10%	289,276	106,202	27,862	7,645
20%	136,586	50,145	13,156	3,610

Table S2: Characteristics of 458 cfDNAs in association with maternal age and gestational age.

Characteristics	cfDNA concentration (ng/ml plasma)[a]			cffDNA fraction (%)[a]		
	Training set (n = 103)	Testing set (n = 355)	*P* value	Training set (n = 103)	Testing set (n = 355)	*P* value
Maternal age, years						
< 35	8.52 (5.87 - 12.48)	7.69 (5.52 - 10.42)	0.1103[b]	11.66 (9.06 - 15.08)	8.82 (6.15 - 12.44)	0.0003[b]
≥ 35	8.22 (6.48 - 10.00)	7.39 (5.91 - 10.23)	0.2972[b]	11.80 (8.86 - 16.35)	8.50 (6.02 - 13.12)	0.0004[b]
P value	0.8010[b]	0.8029[b]		0.7491[b]	0.8743[b]	
Gestational age, weeks						
First trimester (11-13)	6.77 (5.95 - 9.32)	7.95 (4.01 - 9.63)	0.9212[b]	13.13 (10.34 - 15.14)	8.75 (5.98 - 11.10)	0.1333[b]
Second trimester (14-27)	8.60 (6.27 - 12.45)	7.60 (5.69 - 10.40)	0.0361[b]	11.45 (8.93 - 14.86)	8.49 (6.03 - 12.89)	< 0.0001[b]
Third trimester (28-40)	7.61 (5.72 - 8.75)	7.48 (6.17 - 10.31)	0.7925[b]	17.73 (16.88 - 26.04)	11.93 (9.73 - 17.43)	0.0420[b]
P value	0.4249[c]	0.8723[c]		0.0054[c]	0.0373[c]	

cfDNA, cell-free DNA; cffDNA, cell-free fetal DNA.

[a] Values are presented as median (IQR, interquartile range). [b] Mann-Whitney test. [c] Kruskal-Wallis test.

Table S3: Characteristics and test results of 38 testing set subjects with confirmed chromosomal anomalies.

Cases	Demographics		cfDNA profiles		Prenatal screening results			FOS-dPCR-NIPT results				Invasive prenatal diagnostic results	
	MA (y)	GA (w)	YD (ng)	FF (%)	SBT	Ultrasound	NGS-NIPT	Z_{21}	Z_{18}	Z_{13}	Call	CNV-seq	Karyotyping
1	30	18.14	6.72	8.49	NA	Normal	del(18)(q22.2q23)	-0.95	-0.47	0.88	LR	del(18)(q22.2q23) 10040kb P, del(18)(p11.32) 1460kb VUS	46,XX,der(18)(:p11.2→q21.1),t(4;7)(p12;q21.1q22)[17]/45,XX,-18,t(4;7)(p12;q21.1q22)[3]
2	36	18.57	18.44	10.50	NA	Normal	T21	43.82	-1.14	-1.01	T21	dup(21)(q11.2q22.3)	47,XY,+21
3	38	18.00	20.88	15.26	NA	Normal	NA	24.29	-1.20	-1.05	T21	dup(21)(q11.2q22.3)	47,XX,+21
4	41	19.57	49.08	5.68	NA	Normal	T21	11.67	-8.14	-4.62	T21	dup(21)(q11.2q22.3)	47,XX,+21
5	36	19.00	17.16	8.41	NA	Normal	SCA	-0.04	-0.02	6.60	LR	del(Y)(p11.32q12)mos 15%	45,X[5]/46,XY[15]
6	32	21.71	7.08	7.14	NA	Abnormal	LR	-1.16	-1.79	3.40	LR	dup(11)(q23.3q25) 18240kb P, dup(22)(q11.1q11.21) 3480kb P	47,XY,+M
7	27	17.29	53.16	4.15	NA	Normal	SCA	-0.34	1.99	-0.41	LR	del(X)(p22.33q28)mos70%	45,X[15]/46,XX[5]
8	31	13.71	32.04	8.34	NA	Abnormal	NA	0.47	-0.30	-0.76	LR	del(X)(p22.33q28)	45,X
9	34	20.43	30.84	7.98	NA	Normal	SCA	-0.03	1.59	-0.01	LR	dup(X)(p22.3q28)	47,XXX
10	30	22.00	19.61	6.08	NA	Normal	NA	-0.67	0.16	-0.13	LR	del(Y)(p11.32q12)mos 35%	45,X[10]/46,X,+M[10]
11	28	23.00	9.48	11.09	NA	Normal	Abnormal Chr.9	0.00	0.00	0.02	LR	dup(9)(p24.3q34.3)mos 10%	47,XY,+9[5]/46,XY[15]
12	39	17.71	45.24	3.07	NA	Normal	NA	-0.19	1.05	-0.15	LR	seq(1-22)*2,(XX)*1	46,XX,inv(21)(p11.1q21.1)
13	34	19.14	38.88	16.44	NA	Normal	SCA	-0.03	0.11	-0.04	LR	dup(Y)(p11.32q12)	47,XYY
14	32	18.14	35.52	6.10	HR	Normal	NA	0.55	-0.43	-1.66	LR	seq(1-22)*2,(XX)*1	46,XX,t(1;7)(q32;q32)
15	35	17.71	26.88	10.03	NA	Normal	NA	-0.18	-0.25	0.88	LR	seq(1-22)*2,(XY)*1	46,XY,t(4;9)(q35;q22.1)
16	33	17.43	41.52	3.75	NA	Normal	NA	-0.68	2.11	-1.00	LR	del(X)(p21.1) 160kb P	46,XY
17	38	17.43	28.68	8.71	NA	Abnormal	NA	-2.50	28.82	-2.72	T18	dup(18)(p11.32q23)	47,XY,+18
18	33	16.29	42.24	13.10	NA	Normal	T21	41.30	-3.55	-3.08	T21	dup(21)(q11.2q22.3)	47,XY,+21
19	34	17.71	20.41	10.17	NA	Normal	dup(1)(q21.1q21.2)	-1.54	2.37	-0.90	LR	dup(1)(q21.1q21.2) 3400kb P	46,XX
20	36	18.43	27.48	4.59	NA	Normal	T18	-11.70	16.41	-6.60	T18	dup(18)(p11.32q23)	47,XX,+18
21	35	17.86	36.24	7.92	NA	Normal	T21	19.45	-3.68	-2.94	T21	dup(21)(q11.2q22.3)	47,XY,+21
22	42	16.29	21.72	10.28	NA	Abnormal	NA	34.32	-4.38	-4.84	T21	dup(21)(q11.2q22.3)	47,XX,+21
23	29	18.14	2.10	21.86	NA	Normal	T21	132.19	-26.50	-31.55	T21	dup(21)(q11.2q22.3)	47,XY,+21
24	39	18.29	17.88	14.88	NA	Abnormal	NA	42.22	-8.83	-10.66	T21	dup(21)(q11.2q22.3)	47,XY,+21
25	33	12.86	3.02	27.34	NA	Abnormal	NA	38.36	-2.46	-2.56	T21	dup(21)(q11.2q22.3)	47,XX,+21,9qh+
26	27	17.86	13.44	22.55	NA	Abnormal	NA	44.24	-10.87	-13.74	T21	dup(21)(q11.2q22.3)	47,XX,+21
27	43	17.71	17.88	18.60	NA	Normal	NA	73.30	-4.08	-4.20	T21	dup(21)(q11.2q22.3)	47,XX,+21
28	39	28.00	17.28	19.52	NA	Normal	T21	76.73	-2.82	-2.88	T21	dup(21)(q11.2q22.3)	47,XX,+21
29	39	16.43	24.71	15.59	NA	Normal	T21	28.39	-2.42	-2.10	T21	dup(21)(q11.2q22.3)	47,XX,+21

30	31	16.57	20.07	10.87	NA	Normal	T21	31.88	-0.86	-0.75	T21	dup(21)(q11.2q22.3)	47,XY,+21
31	28	16.86	35.73	10.21	NA	Abnormal	NA	56.19	-6.06	-6.54	T21	dup(21)(q11.2q22.3)	47,XY,+21
32	38	18.14	35.50	11.28	NA	Normal	T21	45.34	-3.07	-3.21	T21	dup(21)(q11.2q22.3)	47,XY,+21
33	35	19.43	21.92	15.65	NA	Normal	T21	44.15	-3.34	-3.51	T21	dup(21)(q11.2q22.3)	47,XX,+21
34	39	16.57	22.16	5.87	NA	Abnormal	NA	16.69	-4.33	-3.27	T21	dup(21)(p13q22.3)	47,XY,+21
35	33	19.71	21.69	10.96	HR	Abnormal	NA	61.16	-2.85	-2.54	T21	dup(21)(p13q22.3)	47,XY,+21
36	29	22.86	44.52	8.43	NA	Abnormal	LR	-0.37	-0.14	0.20	LR	seq(1-22)*2,(XY)*1	47,XY,+12[2]/46,XY[18]
37	40	16.71	105.00	5.36	NA	Normal	T13	-1.03	-1.13	41.88	T13	arr(13)*3	NA
38	35	17.00	18.84	9.54	NA	Normal	NA	-4.91	-5.62	48.01	T13	arr(13)*3	NA

MA, maternal age; y, years; GA, gestational age; w, weeks; YD, yield; FF, fetal fraction; SBT, serum biochemical test; NGS-NIPT, next generation sequencing based noninvasive prenatal test; CNV-seq, copy number variation sequencing; NA, not available; LR, low risk; T21, trisomy 21; SCA, sex chromosome aneuploidy; HR, high risk; T18, trisomy 18; T13, trisomy 13; P, pathogenic; VUS, variance of unknown significance.

Table S4: Association of fetal ploidy status with clinical characteristics for 350 testing set subjects

Characteristics	Aneuploidy negative (n = 317)	Aneuploidy positive (n = 33)	*P* value	Trisomy negative (n = 327)	Trisomy positive (n = 23)	*P* value
Maternal age, years						
Median (IQR)	33 (29.5-36)	35 (30.5-38.5)	0.1266[a]	33 (29-36)	36 (33-39)	0.0034[a]
< 35	184 (58.04%)	16 (48.48%)	0.3559[b]	193 (59.02%)	7 (30.43%)	0.0088[b]
≥ 35	133 (41.96%)	17 (51.52%)		134 (40.98%)	16 (69.57%)	
Gestational age, weeks						
Median (IQR)	18.29 (17.14-22.57)	18.14 (16.79-19.50)	0.1053[a]	18.29 (17.29-22.57)	17.86 (16.57-18.43)	0.0170[a]
First trimester (11-13)	6 (1.89%)	2 (6.06%)	0.3111[c]	7 (2.14%)	1 (4.35%)	0.7110[c]
Second trimester (14-27)	302 (95.27%)	30 (90.91%)		311 (95.11%)	21 (91.30%)	
Third trimester (28-40)	9 (2.84%)	1 (3.03%)		9 (2.75%)	1 (4.35%)	
Clinical indications						
Positive SBT[d], (n = 29)	27 (96.43%)	1 (100.00%)	< 0.0001[c]	27 (96.43%)	1 (100.00%)	< 0.0001[c]
Abnormal ultrasound	133 (41.96%)	11 (33.33%)		136 (41.59%)	8 (34.78%)	
Previous affected pregnancy	71 (22.40%)	1 (3.03%)		71 (21.71%)	1 (4.35%)	
Positive NGS-NIPT[d], (n = 66)	16 (34.78%)	18 (90.00%)		22 (40.74%)	12 (100.00%)	

IQR, interquartile range; SBT, serum biochemical test; NGS-NIPT, next generation sequencing based noninvasive prenatal test.

[a] Mann-Whitney test. [b] Fisher's exact test. [c] χ^2 test. [d] SBT and/or NGS-NIPT were not performed for every pregnancy, only pregnancies with reportable results were included.

Table S5: Results of NGS-NIPT and FOS-dPCR-NIPT compared with invasive diagnosis and clinical outcome for 66 testing set plasma samples.

Sample ID	NGS-NIPT results	FOS-dPCR-NIPT results				Invasive prenatal diagnostic results		Clinical outcome
		Z_{21}	Z_{18}	Z_{13}	Call	CNV-seq	Karyotyping	Neonate phenotype
23220919	LR	-0.99	-0.34	0.49	LR	seq(1-22)*2,(XY)*1	46,XY	Normal
23220937	LR	-1.08	0.25	-0.20	LR	seq(1-22)*2,(XY)*1	46,XY	Normal
23220939	LR	-0.06	0.18	-0.10	LR	seq(1-22)*2,(XY)*1	46,XY	Normal
23220943	T3	0.09	-2.53	-0.10	LR	seq(1-22)*2,(XY)*1	46,XY	Normal
23220966	LR	-1.36	-0.72	1.41	LR	seq(1-22)*2,(XY)*1	46,XY	Normal
23221159	del(18)(q22.2q23)	-0.95	-0.47	0.88	LR	del(18)(q22.2q23) 10040kb P,del(18)(p11.32) 1460kb VUS	46,XX,der(18)(:p11.2→q21.1),t(4;7)(p12;q21.1q22)[17]/45,X X,-18,t(4;7)(p12;q21.1q22)[3]	Abortion
23221181	T21	43.82	-1.14	-1.01	T21	dup(21)(q11.2q22.3)	47,XY,+21	Abortion
23221241	T21	11.67	-8.14	-4.62	T21	dup(21)(q11.2q22.3)	47,XX,+21	Abortion
23221280	SCA	-1.32	-0.90	2.63	LR	seq(1-22)*2,(XY)*1	46,XY	Normal
23221285	SCA	-0.04	-0.02	6.60	LR	del(Y)(p11.32q12)mos 15%	45,X[5]/46,XY[15]	Abortion
23221286	del(4)(q21q31)	-0.24	-0.18	0.80	LR	seq(1-22)*2,(XX)*1	46,XX	Normal
23221290	LR	-1.16	-1.79	3.40	LR	dup(11)(q23.3q25) 18240kb P,dup(22)(q11.1q11.21) 3480kb P	47,XY,+M	Abortion
23221326	T13	4.04	-0.86	-1.06	LR	seq(1-22)*2,(XY)*1	46,XY,9ph+	Normal
23221328	SCA	-0.34	1.99	-0.41	LR	del(X)(p22.33q28)mos70%	45,X[15]/46,XX[5]	Abortion
23221333	SCA	-0.03	1.59	-0.01	LR	dup(X)(p22.3q28)	47,XXX	Abortion
23221340	Abnormal Chr.9	0.00	0.00	0.02	LR	dup(9)(p24.3q34.3)mos 10%	47,XY,+9[5]/46,XY[15]	Abortion
23221345	SCA	-0.03	0.11	-0.04	LR	dup(Y)(p11.32q12)	47,XYY	Abortion
23221385	LR	0.45	-1.53	-0.34	LR	seq(1-22)*2,(XY)*1	46,XY	Normal
23221392	LR	1.63	-0.77	-1.37	LR	seq(1-22)*2,(XX)*1	46,XX	Normal
23221393	LR	-0.67	0.40	-0.25	LR	seq(1-22)*2,(XX)*1	46,XX	Normal
23221395	LR	0.38	-0.33	-1.71	LR	seq(1-22)*2,(XX)*1	46,XX	Normal
23221404	LR	-1.07	-0.16	0.17	LR	seq(1-22)*2,(XY)*1	46,XY	Normal
23221408	LR	-0.11	2.11	-0.13	LR	seq(1-22)*2,(XY)*1	46,XY	Normal
23221412	LR	-6.03	0.84	-0.72	LR	seq(1-22)*2,(XX)*1	46,XX	Normal
23221416	T21	41.30	-3.55	-3.08	T21	dup(21)(q11.2q22.3)	47,XY,+21	Abortion
23221421	LR	1.56	-0.89	-1.90	LR	dup(4)(q35.1q35.2) VUS	46,XX	Normal
23221428	dup(1)(q21.1q21.2)	-1.54	2.37	-0.90	LR	dup(1)(q21.1q21.2) 3400kb P	46,XX	Abortion
23221429	dup(22)(q11.22q12.1)	-0.14	-0.12	0.86	LR	dup(22)(q11.22q11.23) 2000kb VUS	46,XX	Normal
23221432	SCA	0.47	-0.25	-0.15	LR	seq(1-22)*2,(XX)*1	46,XX	Normal
23221433	LR	0.63	-0.30	-0.56	LR	seq(1-22)*2,(XY)*1	46,XY	Normal

23221477	T18	-11.70	16.41	-6.60	T18	dup(18)(p11.32q23)	47,XX,+18	Abortion
23221588	T21	19.45	-3.68	-2.94	T21	dup(21)(q11.2q22.3)	47,XY,+21	Abortion
23230137	T21	132.19	-26.50	-31.55	T21	dup(21)(q11.2q22.3)	47,XY,+21	Abortion
23230218	T21	76.73	-2.82	-2.88	T21	dup(21)(q11.2q22.3)	47,XX,+21	Abortion
23230221	T21	28.39	-2.42	-2.10	T21	dup(21)(q11.2q22.3)	47,XX,+21	Abortion
23230268	T21	31.88	-0.86	-0.75	T21	dup(21)(q11.2q22.3)	47,XY,+21	Abortion
23230314	T21	45.34	-3.07	-3.21	T21	dup(21)(q11.2q22.3)	47,XY,+21	Abortion
23230360	T21	44.15	-3.34	-3.51	T21	dup(21)(q11.2q22.3)	47,XX,+21	Abortion
23230372	LR	-0.55	1.65	-0.39	LR	seq(1-22)*2,(XY)*1	46,XY	Normal
23230391	LR	-0.70	1.23	-1.62	LR	seq(1-22)*2,(XX)*1	46,XX	Normal
23230406	LR	-0.12	-0.01	0.01	LR	seq(1-22)*2,(XX)*1	46,XX	Normal
23230408	LR	-2.50	-0.59	0.71	LR	seq(1-22)*2,(XX)*1	46,XX	Normal
23230410	LR	-0.18	0.13	-0.07	LR	seq(1-22)*2,(XY)*1	46,XY	Normal
23230411	LR	-0.24	-0.30	1.42	LR	seq(1-22)*2,(XX)*1	46,XX	Normal
23230412	LR	-0.42	1.07	-0.69	LR	seq(1-22)*2,(XX)*1	46,XX	Normal
23230433	LR	-0.21	-0.37	0.51	LR	seq(1-22)*2,(XY)*1	46,XY	Normal
23230490	T21	-1.47	-0.35	0.42	LR	seq(1-22)*2,(XX)*1	46,XX	Normal
23230505	SCA	1.26	-1.80	-0.72	LR	seq(1-22)*2,(XY)*1	46,XY	Normal
23230529	LR	-0.23	-0.40	0.55	LR	seq(1-22)*2,(XY)*1	46,XY	Normal
23230547	LR	0.57	-0.18	-0.26	LR	seq(1-22)*2,(XY)*1	46,XY	Normal
23230551	LR	-0.15	-0.97	0.17	LR	seq(1-22)*2,(XX)*1	46,XX	Normal
23230557	SCA	-0.02	0.01	-0.49	LR	seq(1-22)*2,(XX)*1	46,XX	Normal
23230571	T13	-0.86	-0.55	1.44	LR	seq(1-22)*2,(XX)*1	46,XX	Normal
23230598	SCA	0.03	-0.03	-0.21	LR	seq(1-22)*2,(XY)*1	46,XY	Normal
23230632	T9	-1.22	-0.41	0.57	LR	seq(1-22)*2,(XX)*1	46,XX	Normal
23230674	LR	-0.22	-0.05	0.05	LR	seq(1-22)*2,(XX)*1	46,XX	Normal
23230687	LR	-0.37	-0.14	0.20	LR	seq(1-22)*2,(XY)*1	47,XY,+12[2]/46,XY[18]	Abortion
23230691	LR	-0.19	-0.23	2.05	LR	seq(1-22)*2,(XX)*1	46,XX	Normal
23230692	LR	0.84	-0.65	-0.35	LR	seq(1-22)*2,(XY)*1	46,XY	Normal
23230693	LR	0.42	-0.08	-0.06	LR	seq(1-22)*2,(XY)*1	46,XY	Normal
23230695	LR	-0.22	1.31	-0.18	LR	seq(1-22)*2,(XY)*1	46,XY	Normal
23230701	dup(16)(q11.2q23.2)	-0.68	1.16	-0.41	LR	seq(1-22)*2,(XX)*1	46,XX	Normal
23230702	LR	-0.22	-0.52	0.38	LR	seq(1-22)*2,(XY)*1	46,XY	Normal
23230704	SCA	-0.30	-0.07	0.09	LR	seq(1-22)*2,(XX)*1	46,XX	Normal

23230720	SCA	-0.06	0.05	-0.03	LR	seq(1-22)*2,(XX)*1	46,XX	Normal
27230134	T13	-1.03	-1.13	41.88	T13	arr(13)*3	NA	Abortion

NGS-NIPT, next generation sequencing based noninvasive prenatal test; CNV-seq, copy number variation sequencing; LR, low risk; T3, trisomy 3; T21, trisomy 21; SCA, sex chromosome aneuploidy; T13, trisomy 13; T18, trisomy 18; T9, trisomy 9; NA, not available; P, pathogenic; VUS, variance of unknown significance.

Table S6: Diagnostic performance of FOS-dPCR-NIPT and NGS-NIPT for the indicated aneuploidy in 66 testing set plasma samples.

Aneuploidy identified by	True ploidy status confimed by invasive diagnosis and clinical outcome									
	Trisomy 21		Trisomy 18		Trisomy 13		All three trisomies		All aneuploidies	
**FOS-dPCR-NIPT**	Positive	Negative	Positive	Negative	Positive	Negative	Positive	Negative	Positive	Negative
Positive	10	0	1	0	1	0	12	0	12	0
Negative	0	56	0	65	0	65	0	54	8	46
**NGS-NIPT**	Positive	Negative	Positive	Negative	Positive	Negative	Positive	Negative	Positive	Negative
Positive	10	1	1	0	1	2	12	3	18	14
Negative	0	55	0	65	0	63	0	51	2	32

Diagnostic performance	Trisomy 21 detection	Trisomy 18 detection	Trisomy 13 detection	Combined trisomy detection	Combined aneuploidy detection
**FOS-dPCR-NIPT**					
AUC (95% CI)	1.000 (0.946-1.000)	1.000 (0.946-1.000)	1.000 (0.946-1.000)	1.000 (0.946-1.000)	0.800 (0.683-0.888)
Sensitivity, % (95% CI)	100.00 (69.15-100.00)	100.00 (2.50-100.00)	100.00 (2.50-100.00)	100.00 (73.54-100.00)	60.00 (36.05-80.88)
Specificity, % (95% CI)	100.00 (93.63-100.00)	100.00 (94.48-100.00)	100.00 (94.48-100.00)	100.00 (93.40-100.00)	100.00 (92.29-100.00)
PPV, % (95% CI)	100	100	100	100	100
NPV, % (95% CI)	100	100	100	100	85.19 (77.07-90.77)
Accuracy, % (95% CI)	100.00 (94.56-100.00)	100.00 (94.56-100.00)	100.00 (94.56-100.00)	100.00 (94.56-100.00)	87.88 (77.51-94.62)
**NGS-NIPT**					
AUC (95% CI)	0.991 (0.929-1.000)	1.000 (0.946-1.000)	0.985 (0.918-1.000)	0.972 (0.899-0.997)	0.798 (0.681-0.887)
Sensitivity, % (95% CI)	100.00 (69.15-100.00)	100.00 (2.50-100.00)	100.00 (2.50-100.00)	100.00 (73.54-100.00)	90.00 (68.30-98.77)
Specificity, % (95% CI)	98.21 (90.45-99.95)	100.00 (94.48-100.00)	96.92 (89.32-99.63)	94.44 (84.61-98.84)	69.57 (54.25-82.26)
PPV, % (95% CI)	90.91 (58.91-98.59)	100	33.33 (11.33-66.18)	80.00 (57.12-92.32)	56.25 (44.78-67.08)
NPV, % (95% CI)	100	100	100	100	94.12 (80.91-98.37)
Accuracy, % (95% CI)	98.48 (91.84-99.96)	100.00 (94.56-100.00)	96.97 (89.48-99.63)	95.45 (87.29-99.05)	75.76 (63.64-85.46)

NGS-NIPT, next generation sequencing based noninvasive prenatal test; AUC, area under the receiver operator characteristic curve; PPV, positive predicative value; NPV, negative predicative value; CI, confidence interval.

Table S7: Variables in the cost-effectiveness model.

Variables	Value
Epidemiology, %	
Percentage of pregnant women ≥ 35 years[a]	15
Incidence of T21 in pregnant women[1]	
< 35 years	0.1243
≥ 35 years	0.2857
Overall	0.1485
PRL rate[2]	0.3
T21 pregnancy termination rate[a]	95
T21 spontaneous miscarriage rate[3]	25
Non-T21 spontaneous miscarriage rate[4]	15.3
Screening performance, sensitivity (%)/specificity (%)	
SBT+NT (cut-off: 1/1000)[5]	79/96
NGS-NIPT[6]	99.5/100
FOS-dPCR-NIPT (maximum sensitivity)[b]	100/98.89
FOS-dPCR-NIPT (maximum specificity)[b]	82.35/100
Screening uptake rate[a], %	
SBT+NT	75
NIPT as 1st screen	80
NIPT as 2nd screen	95
Invasive diagnosis uptake rate[a], %	
In NIPT positive pregnant women	100
In pregnant women ≥ 35 years	80
Direct cost (base)[a], $	
SBT+NT	35
NGS-NIPT	200
FOS-dPCR-NIPT	100
Invasive diagnosis	800
Pregnancy termination	385
PRL	480
Spontaneous abortion	150
Natural labor	1,035
Indirect cost (base)[c], $	
During screen	40
During diagnosis	40
During childbirth	130

T21, trisomy 21; PRL, procedure-related fetus loss caused by invasive diagnosis; SBT, serum biochemical test; NT, nuchal translucency; NGS-NIPT, next generation sequencing based noninvasive prenatal test.

[a] Data are from local official statistics. [b] Screening performance of dPCR-NIPT for T21 is estimated from the current study with the low end of 95% confidence interval (CI) for specificity at maximum sensitivity of 100%, or with the low end of 95% CI for sensitivity at maximum specificity of 100%, respecitively. [c] Indirect cost is from local official statistics, which includes lost earnings for pregnant women during screen/diagnosis and cost for caregivers during childbirth.

References

1. Mai CT, Isenburg JL, Canfield MA, et al. National population-based estimates for major birth defects, 2010-2014. *Birth Defects Res* 2019; **111**(18): 1420-35.
2. Salomon LJ, Sotiriadis A, Wulff CB, Odibo A, Akolekar R. Risk of miscarriage following amniocentesis or chorionic villus sampling: systematic review of literature and updated meta-analysis. *Ultrasound in Obstetrics & Gynecology* 2019; **54**(4): 442-51.
3. Savva GM, Morris JK, Mutton DE, Alberman E. Maternal age-specific fetal loss rates in Down syndrome pregnancies. *Prenatal Diagnosis* 2006; **26**(6): 499-504.
4. Quenby S, Gallos ID, Dhillon-Smith RK, et al. Miscarriage matters: the epidemiological, physical, psychological, and economic costs of early pregnancy loss. *The Lancet* 2021; **397**(10285): 1658-67.
5. Tu S, Rosenthal M, Wang D, Huang J, Chen Y. Performance of prenatal screening using maternal serum and ultrasound markers for Down syndrome in Chinese women: a systematic review and meta-analysis. *BJOG: An International Journal of Obstetrics & Gynaecology* 2016; **123**(S3): 12-22.
6. Jin J, Yang J, Chen Y, Huang J. Systematic review and meta-analysis of non-invasive prenatal DNA testing for trisomy 21: implications for implementation in China. *Prenatal Diagnosis* 2017; **37**(9): 864-73.
